# Disability sport profile of Ghana: evolution, policies, politics and participation barriers

**DOI:** 10.3389/fspor.2025.1645536

**Published:** 2025-08-25

**Authors:** Derrick Charway, Dennis Osei-Nimo Annor, Davies Banda

**Affiliations:** 1 Department of Sport and Social Sciences, Norwegian School of Sport Sciences, Oslo, Norway; 2 Health and Physical Cultures for Social Justice, Western University, London, ON, Canada; 3 Moray House School of Education and Sport, ISPEHS, The University of Edinburgh, Edinburgh, United Kingdom

**Keywords:** ableism, barriers, Ghana, mainstreaming disability sport, policy implementation

## Abstract

The profile analyses the landscape of disability sport in Ghana, tracing its historical evolution and contemporary challenges. Alongside legislative advancements and the dedication of various stakeholders, an increase in the persons with disability population has been observed. Based on data from the Ghana Statistical Service census, this demographic rose from 737,743 in 2010 to 2,098,138 in 2021, constituting 3% and 8% of the Ghanaian population in those respective years. Nevertheless, significant barriers to the mainstreaming of disability sport persist. The analysis delves into the interplay of cultural norms, government policies, and collaborative efforts in shaping the trajectory of disability sport in the country. Insights into the population of persons with disabilities and their engagement in sport offer a foundation for discussion. Further, an analysis of the roles of key state and non-state organisations, alongside international partners, emphasises the need to move from symbolic implementation to genuinely inclusive implementation. Contemporary issues such as political infighting, inadequate funding, gender dynamics, limited media coverage, exploitation of disability sport and systemic neglect continue to hinder progress. The profile underscores the urgent need for sustained policy implementation, increased investment, and a more inclusive and collaborative approach to secure a promising future for disability sport in Ghana.

## 1 Introduction

A simple Google search on disability sport in Ghana often reveals troubling reports of exploitation of para-athletes and disability sport by various sport governing bodies, including state institutions. One recent high-profile case that drew global attention was a 23rd July 2024 British Broadcasting Corporation (BBC) report titled “Ghana investigating para-athletic ‘imposters’ in Norway” (1). While such headlines undoubtedly tarnish the public image of disability sport in Ghana, they do not capture the full complexity of the situation. Research detailing disability sport (also referred to as para-sport) in Ghana has been limited. The few empirical studies available reveal significant gaps between disability sport policy and its implementation, alongside prevalent ableism, a lack of financial support, and persistent governance issues (2, 3). All these factors have considerable implications for the development and sustainability of disability sport in the country. These concerning issues resonate with the broader global discourse surrounding disability sport, as captured in various scholarly publications (4–6). Consequently, the practical and authentic implementation of disability sport remains elusive, and Ghana is no exception. To critically engage with the current profile of disability sport in Ghana, it is essential first to revisit the socio-cultural and structural experiences of persons with disabilities in both precolonial and postcolonial contexts. Such a historical overview provides a quintessential foundation for understanding the present state of disability sport and proposing meaningful, context-specific recommendations to support its development.

## 2 Historical foundations

### 2.1 Pre-colonial era

The traditional lore and treatment of persons with disabilities in precolonial Ghana (comprising West African nations before the 15th century) were deeply embedded in cultural, spiritual, and social paradigms, varying significantly across ethnic groups. Barclay (7), stated that “each society possessed its own unique cultural lens through which individuals read and understood numerous forms of embodied differences.” For instance, the Ashantis and Gonjas of present-day Ghana associated disability with social unfitness, and often excluding individuals with physical or cognitive impairments from political authority or subjecting them to abandonment (8, 9). In contrast, cultural beliefs among the Ga-Adangbe of Ghana, often regarded intellectual disability as a sign of divine presence, with affected individuals treated with reverence (7, 10). In other cultures, like the Igbo and Yoruba of present-day Nigeria, individuals with certain impairments were integrated into society based on their abilities, serving as oral historians, musicians, craftsmen, or healers (7). One enduring characteristic across the diverse ethnic groups of precolonial West Africa was the spiritualisation of disability, often construed as a sign of imbalance, divine punishment, or the influence of supernatural forces. For instance, the notions of “spirit children” in Ghana and “snake children” in Côte d'Ivoire are associated with children born with impairments, who were subjected to ritual abandonment or death (11). These early cultural logics established both the exclusionary tendencies and the adaptive potential that continue to inform disability discourse in contemporary Ghana.

### 2.2 Colonial era

The British colonial era in Ghana (1874–1957) fundamentally reshaped the understanding and treatment of persons with disabilities through the imposition of administrative, legal, and sociocultural frameworks. British rule predominantly adopted a biomedical and administrative lens, categorising persons with disabilities as unproductive and dependent, thereby reinforcing their marginalisation within legal and policy structures (12). Colonial economic shifts, such as urbanization and wage labour, further excluded persons with disabilities who were unable to participate in formal employment, increasing their economic vulnerability. The arrival of Western education, Christian missionary efforts, and modern medical practices introduced new ideologies that often displaced indigenous understandings of disability. Western paradigms framed disability primarily as an individual medical deficiency, rather than a communal or spiritual condition, as had been previously common in many Ghanaian societies (13). Early integration of sport within these Western institutions was primarily therapeutic rather than empowering. Institutionalisation, spearheaded by missionary efforts (resulting in the establishment of the Akropong School for the Blind, founded in 1945, and Ghana Mission School for the Deaf, founded in 1957), introduced Western physical education focused on rehabilitation (14, 15). These efforts introduced adapted sport like goalball and sitting volleyball were employed within a paternalistic care model. While limited in scope and focused on therapy, these early interventions laid the groundwork for the future development of disability sport in Ghana as a potential avenue for inclusive physical activity, disability rights, empowerment and social change.

### 2.3 Post-colonial era

In the postcolonial era following Ghana's independence in 1957, disability-related issues, particularly in sport, remained peripheral to national development priorities. While Kwame Nkrumah's (the first president of Ghana) government emphasised national unity and international recognition through mainstream sport, disability sport was notably absent from policy agendas. During the early period of independence (1957–1980s), colonial legacy structures were still in place. For instance, the Department of Social Welfare, established in 1946, began offering basic support services to persons with disabilities. This effort was complemented by faith-based and voluntary organisations, such as the Salvation Army, founded in 1922, which established homes and training centres to aid persons with disabilities (16). Furthermore, advocacy groups like the Ghana Society for the Blind (founded in 1951), Ghana Association of the Blind (founded in 1963) and the Ghana National Association of the Deaf (founded in 1968) were also established and would later become instrumental in shaping disability (sport) policy and awareness. A further significant milestone in Ghana's disability sport landscape can be traced back to Kwame Nkrumah's 1960 consultations with Sir John Wilson, a notable British disability policy expert who, at the time, was the Secretary of the Royal Commonwealth Society for the Blind (17). This pivotal consultation culminated in the Wilson Committee Report of 1960. The Report played a formative role in shaping Ghana's Education Act, 1961 (Act 87) and approach to inclusive education and disability services. Specifically, the Report findings led to the establishment of special basic schools for persons with disabilities and facilitated their integration into mainstream public secondary and tertiary educational institutions, including teacher training colleges and universities—this is captured in Section 7(b) of the Education Act, 1961 (Act 87). This progressive initiative has, to date, ensured that adapted and inclusive physical education remains an integral component of the Ghana Education Service's teaching curriculum, thereby embedding principles of accessibility and inclusion within the nation's educational framework and, by extension, within the foundations of disability sport.

However, disability sport remained informal, largely confined to rehabilitation-oriented activities within institutions such as the Nsawam Orthopaedic Training Centre (founded in 1961) and Accra Rehabilitation Centre (founded in 1962). These programmes, typically supported by Non-governmental Organisations (NGOs) and churches, lacked competitive orientation and national policy integration. Additionally, the broader framing of disability sport during the early independence period was grounded in charity and care, rather than empowerment or rights-based inclusion. The progress achieved during the early years of Ghana's independence was significantly undermined by a series of coup d'états and counter-coups, which ushered in successive military regimes—notably the National Liberation Council (1966–1969), the National Redemption Council (1972–1975), and the Supreme Military Council (1975–1979). As with many post-independence African nations under military rule, Ghana experienced political instability and economic volatility, which severely disrupted the development of disability sport programmes in schools and curtailed the activities of NGOs supporting persons with disabilities. This regression occurred during a period, according to Novak (18) “when tremendous advances in technology and human development were occurring in disability sport.” It is worth noting that although brief periods of democratic governance occurred (from 1969 to 1972 and 1979–1981), these were insufficient to remedy the deteriorating conditions given the broader economic fragility and volatile climate.

In the late 1980s, Ghana acceded to the World Bank and the International Monetary Fund's neoliberal reforms, namely the Economic Recovery Programme (ERP) and Structural Adjustment Programme (SAP), which also necessitated a transition to democratic governance. Although the ERP and SAP policies resulted in further reductions to social welfare provisions, they simultaneously stimulated the emergence of a nascent disability rights movement (2). As Charway and Houlihan (19) observe, several prominent non-state actors in the 1980s became “keen advocates for para-sport, para-athlete rights to play, and education against ableism in Ghana.” These included the Ghana Federation of Disability Organisations, the Ghana Society of the Physically Disabled, and the Danish Sports Organisation for the Disabled (DSOD, now Parasport Denmark). Following the dire economic and political climate of the late 1980s, Ghana returned to multi-party democracy and the rule of law on 7th January 1993. As Charway (20) observes in his review of the *History and Politics of Sport-for-Development*, the “incorporation of social capital into neoliberal policies led to the establishment of civil societies/NGOs that mobilise social resources [including (disability) sport] for development.” It is therefore not surprising that upon Ghana's return to multi-party democracy in 1993, the National Sport Policy of 1994 was drafted with explicit inclusive provisions. This was a crucial step to enhance and provide depth to the then-existing Sports Act Supreme Military Council Decree (SMCD) 54 of 1976, which notably contained no specific provisions for disability sport. The 1994 policy thus marked a significant shift towards formally integrating disability sport within the national sporting agenda, reflecting broader societal changes and the growing recognition of civil society's role in development.

The period spanning the 1990s and 2000s witnessed a significant shift in disability sport in Ghana, transitioning gradually from charity-based, recreational focus and rehabilitative models toward structured, competitive participation at national and international levels. This period, often marked as the emergence of organised disability sport, was shaped by both global disability rights movements and local advocacy efforts aimed at redefining sport as a domain of empowerment rather than passive care (15). During the 1990s, Ghana's engagement with international disability frameworks increased, leading to the formalisation of national structures. The Ghana Society of the Physically Disabled began organising community-level sporting events, laying the groundwork for broader institutional coordination. A pivotal milestone was the establishment of the Association of Sports for the Disabled (ASFOD) by the National Sports Policy of 1994 and thence the National Paralympic Committee of Ghana (NPC-Ghana) in the late 1990s, which provided the organisational infrastructure to support elite disability sport (19). This initiative culminated in Ghana's debut at the summer Paralympic Games in Athens in 2004, with athletes competing in athletics (track and field) and powerlifting. The athletics contingent comprised Nkegbe Botsyo, who contested the men's 100 m, 200 m, and 400 m events within the T54 classification, alongside Ajara Mohammed, competing in the women's 800 m and marathon, also in the T54 category. In powerlifting, Alfred Adjetey Sowah represented Ghana in the men's up to 52 kg division. Fast forward to 2024, Ghana dispatched four athletes to the Paralympic Games in Paris, competing across an expanded range of disciplines. The delegation included participants in athletics, with Tahiru Haruna competing in the men's +107 kg powerlifting event and Zinabu Issah in the women's Shot Put F57 and Discus Throw F57. Para-cycling saw Frederick Assor participate in the men's time trial B and pursuit B. Furthermore, Patricia Kyeremaa represented Ghana in taekwondo, contesting the women's +65 kg category. Whilst there was no significant difference in the number of athletes between the 2004 and 2024 campaigns, a relatively gender balance and higher media coverage was observed ahead of the 2024 Paris Summer Paralympics.

In the present era, institutional development of disability sport has paralleled the burgeoning visibility from the 1990s and 2000s. The establishment of federations such as the Ghana Wheelchair Basketball Federation, the Ghana Amputee Football Federation, and the Ghana Blind Sports Association reflects a diversification of sport offerings and the integration of disability sport into national sport governance structures, notably through initiatives within the National Sports Authority and the Ministry of Sport and Recreation. Additionally, the sustained contributions of international bodies such as the International Olympic Committee (IOC), the International Paralympic Committee (IPC), and the United Nations (UN) have been instrumental in supporting the development of disability sport in Ghana. Notably, a range of UN-led reforms have laid important foundations for the mainstreaming of disability sport. These include, but are not limited to, initiatives on the Sport for Development and Peace agenda (previously spearheaded by the defunct UN Office on Sport for Development and Peace) as well as the broader global development goals underpinned by the Millennium Development Goals (MDGs) and the Sustainable Development Goals (SDGs). Equally significant is the UN Convention on the Rights of Persons with Disabilities (UN-CRPD) signed in 2007 (and ratified in 2012), which provides a comprehensive rights-based framework for the inclusion of persons with disabilities, including in sport and physical activity. Furthermore, UNICEF has played a key role in implementing inclusive sport for development programmes in Ghana, often in collaboration with the Ghana Education Service (21). These partnerships highlight a multifaceted approach, combining international advocacy and policy with practical, on-the-ground initiatives to foster the mainstreaming and legal integration of disability sport within Ghana. The subsequent section will examine the evolution of the legal frameworks and governing structures of disability sport in Ghana.

## 3 Governing structures

Ghana's 2021 census revealed 2,098,138 peosons with disabilities, representing 8% of the population (22). This is a significant increase from 3% in 2010. The Ghana Statistical Service (22) reported that the prevalence of disability is higher among females (8.8%) than males (6.7%) and further revealed that disabilities are more prevalent in rural areas (9.5%) than in urban areas (6.5%). Despite these figures, the precise participation figures for disability sport remain elusive, reflecting a broader lack of national data on sport engagement. Nevertheless, a dedicated policy community, comprising the Ministry of Sports and Recreation, National Sports Authority, ASFOD, and the NPC-Ghana, collectively governs and implements disability sport initiatives in the country.

The Ministry of Sports and Recreation serves as both a policy driver and coordinating body for disability sport in Ghana. Its pivotal role in development and mainstreaming is enshrined in the Sports Act, 2016 (Act 934), which mandates the inclusion of persons with disabilities in national sport development. The Ministry's policy objectives, outlined in its Medium-Term Expenditure Frameworks, align with global development goals like the SDGs, promoting sport as a tool for youth development, health, and social inclusion (19). It actively works to provide disability-friendly facilities, support skill development for disabled youth, and foster community-level collaboration with disability groups. Through the National Sports Authority and the District Sports Units, the Ministry facilitates the implementation of inclusive sport policies at the grassroots. These Sports Units, often funded in part by the District Assemblies Common Fund, are crucial for delivering sport programmes, including disability sport, to local communities. Collaboration with other key stakeholders, such as the Ministry of Education, Ghana Education Service, and various disability sport federations and civil society organisations, ensures a comprehensive approach to mainstreaming disability sport in Ghana.

The National Sports Authority acts as one of the Ministry's key policy and implementation arms, with a representative from the NPC-Ghana on its governing board. The National Sports Authority operates regional and district structures nationwide to promote sport and physical activities, leveraging sport for broader social benefits. As an implementing body, it collaborates with various national sport associations and federations to develop sport nationally and facilitate participation in domestic and international competitions. Currently, disability sport disciplines registered with the National Sports Authority include the Ghana Amputee Football Association, Ghana Blind Sport Association, Ghana Deaf Sports Federation, and the NPC-Ghana. Notably, several disability sport associations, such as the Ghana Blind Sport Association and Ghana Deaf Sports Federation, run regional sport-wings that actively collaborate with community organisations to promote their respective sport and empower persons with disabilities at grassroots level.

As previously mentioned, ASFOD emerged from the inclusive provisions of the 1994 National Sport Policy. It was originally tasked with organising disabled sports groups, whether Paralympic or not, and advocating for the rights of persons with disabilities to participate in sport and physical activity. Now operating as a non-state organisation, ASFOD's role has significantly diminished since the growing prominence of NPC-Ghana from the early 2000s. Furthermore, ASFOD's omission from the National Sports Act 2016 (Act 934), along with its absence from the governing board of the National Sports Authority, poses a threat to its continued relevance. However, some disability sport federations not affiliated with the NPC-Ghana argue that ASFOD should be recognised as a consolidating body for all disabled sports associations, whereas others believe it should primarily act as an advocacy group.

Having begun operating in the late 1990s, NPC-Ghana achieved full organisational status in 2003 (19). Unlike ASFOD, NPC-Ghana is federated under the IPC and serves as the central body for promoting and developing Paralympic sport in Ghana. NPC-Ghana functions as an independent entity committed to fostering inclusivity and excellence in sport for persons with disabilities. Its establishment was significantly supported by early advocacy groups such as the Ghana Society for the Physically Disabled, Ghana Blind Union, Ghana National Association for the Deaf, Ghana Federation of Disability Organisations, Ghana Society of the Physically Disabled, and Parasport Denmark.

While the aforementioned organisations play pivotal roles, the instrumental contributions of other sectors are crucial. The media, Civil Society Organisations (including religious bodies), philanthropists, and corporate Ghana have been vital in funding and supporting disability sport initiatives across the nation.

## 4 Framing disability sport in Ghana: social and policy dimensions

As previously discussed, disability sport in Ghana, while not formally institutionalised until the 1990s, has evolved through a series of distinct models or frameworks: the social, moral and medical. In the pre-colonial period, the social model of disability was prevalent in communities that embraced it, characterised by communal support for persons with disabilities and their active participation in social activities like drumming or singing, tailored to their specific abilities. Also associated with this era is the moral model. This model defined disability through prevailing spiritual or mythological narratives, leading to both negative and positive perceptions about persons with disability (23). Negatively, disability was sometimes viewed as a form of deviance or punishment, while positively, certain conditions were seen with reverence, eliciting sympathy or even spiritual veneration. The medical model, which emerged during the colonial period, became more prominent after the Second World War to address the needs of injured soldiers (24). This approach shifted the focus toward rehabilitation and treatment, with government agencies and non-governmental organisations, especially religious institutions, playing significant roles in Ghana. One of the key aspects of this model was the use of customised training and physical fitness programmes to rehabilitate persons with disabilities.

It is noteworthy that whilst the aforementioned three models are not entirely obsolete in the current era, the institutional model has undeniably gained considerable prominence in Ghana, like most parts of the world. This model views disability sport through institutional frameworks and policy mechanisms, positioning it increasingly as a form of organised or association-based sport. Emerging in the postcolonial era, the institutional model marked a gradual yet significant shift toward structured inclusion. This model saw the integration of persons with disabilities in formally organised physical activities within schools and sport federations, progressively leading to the establishment of disabled sport associations across Ghana. This, in turn, became the precursor for the wider development of disability sport. Despite these significant contributions to the growth of disability sport and the formation of dedicated associations in Ghana, it is crucial to note that there is currently no standalone disability sport policy. Instead, the legal framework for disability sport is fragmented, being dispersed across various national laws and policies. We will now proceed to analyse these relevant national policies/statutes and corresponding stakeholders to illustrate how disability sport is legally underpinned in Ghana.

## 5 Current legislative developments: implications for disability sport

### 5.1 Education acts

The most recent and comprehensive legislative reform in Ghana's education sector is the enactment of the Pre-Tertiary Education Act, 2020 (Act 1049), which received presidential assent on 29th December 2020. This statute repeals and consolidates several key legislative instruments, namely, the Education Act, 2008 (Act 778); the Ghana Education Service Act, 1995 (Act 506); and the Education Act, 1961 (Act 87). Thus, effectively overhauls the legal framework governing pre-tertiary education in the country. The overarching objective of Act 1049 is to establish a comprehensive, inclusive, and decentralised education system, aiming to cultivate individuals equipped with the essential knowledge, skills, and values required for active and productive citizenship, thereby fostering national development. Section 1, subsections (a) and (b), affirms the establishment of education services extending from basic to senior high school levels. Of particular relevance is Section 5, titled “Inclusive Education”, which comprises five subsections dedicated to the advancement of inclusive and environmentally accessible special education. In addition, several other provisions, specifically Sections 36(2) and (3), 51(g), 52, 58(h), 78(2) and (3), 93(b), and 94, reinforce the importance of special education, including adapted physical education and sport, particularly at the basic and secondary school levels. To support the broader objectives of education services, Section 9(f) of the Act mandates the promotion of “sports and cultural activities in schools in collaboration with the appropriate institutions and authorities.” This explicitly integrates physical education and sport, including disability sport, into the national education framework.

The implementation and governance of Act 1049 fall under the Ministry of Education, with administrative oversight provided by the Education Service, which is a body corporate established by Act 1049. The Education Service operates a decentralised structure encompassing regional and district offices, enabling it to work in close collaboration with Ghana's 261 metropolitan, municipal, and district assemblies (MMDAs—also called District Assemblies), under the Ministry of Local Government and Rural Development. Further cooperation is also established with Faith-Based Organisations, many of which manage both mainstream and special schools across the country. Although not explicitly codified within the Act 1049, functional collaborations frequently occur at the district level with other relevant bodies such as the Ministry of Sports and Recreation, and various disabled sport federations or associations, particularly those with grassroots structures embedded in local communities (3). In addition to Act 1049, the Education Regulatory Bodies Act, 2020 (Act 1023) was enacted to streamline the regulation of education in Ghana. This legislation strengthens institutional oversight to ensure quality assurance, accreditation, and standardisation across all tiers of education. Notably, Section 4(c) of the Act, on the Ghana Tertiary Education Commission (GTEC), and Section 90(1)(b), relating to the National Schools Inspectorate Authority (NaSIA), contain inclusive mandates. The GTEC and NaSIA mandates promote affirmative action for persons with disabilities and require the inclusion of special education professionals on relevant governing boards, respectively.

Given the legislative emphasis on inclusive and holistic education of the two Acts (1049 and 1023), it is not surprising that a significant number of Ghana's national para-athletes are identified through the basic and tertiary education systems. These frameworks collectively illustrate the strategic role of education policy in mainstreaming disability sport and promoting inclusive national development.

### 5.2 Local government acts

Under the purview of the Ministry of Local Government and Rural Development, Ghana has enacted several significant governmental statutes, both historically and in the present, which contain inclusive provisions vital for the advancement of disability sport. Among these, three statutes are particularly prominent: the Local Governance Act, 2016 (Act 936), which establishes the District Assemblies and mandates the operation of the Department of Social Welfare and Community Development (DSWCD); the Local Government Service Act, 2003 (Act 656), which governs the administrative and human resource functions of local government institutions; and the District Assemblies Common Fund (DACF) Act, 1993 (Act 455), which allocates financial resources to support the operations of District Assemblies.

The core functions of District Assemblies relevant to persons with disabilities (and by extension, disability sport) include infrastructure development, social welfare service delivery (facilitated in collaboration with the DSWCD), financial support via the DACF, and participatory governance that promotes community engagement, including the organisation of disability sport initiatives. The DSWCD plays a central role by coordinating community-based rehabilitation programmes for persons with disabilities, promoting access to social welfare services for marginalised populations, and facilitating partnerships between NGOs and communities in the delivery of social interventions. Importantly, DSWCD offices across the country often provide physical spaces (such as offices and meeting venues) for various disability sport federations and associations, thereby serving as an infrastructural base for disability sport at the local level. In parallel, the DACF has proven instrumental in supporting social and sporting activities for persons with disabilities through its annual allocations, including funding directed to District Sports Units, which will be discussed further in the context of the National Sports Act, 2016 (Act 934).

Although the District Assemblies and the DSWCD are recognised as central stakeholders, a broader ecosystem of actors indirectly contributes to disability sport governance and implementation. These include the Parliament of Ghana, which approves annual DACF allocations; the media, which shapes public discourse on inclusivity and disability sport; the Ministry of Sports and Recreation, which oversees national sporting frameworks; disability sport federations and associations, which champion disability sport; and civil society organisations, which promote the empowerment, health, and social inclusion of persons with disabilities.

### 5.3 The persons with disability act 2006 (act 715)

The Persons with Disability Act, 2006 (Act 715) of Ghana provides a legislative framework aimed at promoting and protecting the rights of persons with disabilities. Although the Act does not explicitly mention “disability sport” in detail, several of its provisions have strong implications for the development and promotion of sport and physical activity among persons with disabilities. Notably, Section 17 mandates the state to ensure the establishment of special schools and free education for persons with disabilities. Additionally, Section 21 tasks the Ministry of Education with ensuring that education for persons with disabilities includes the opportunity for recreational and sporting activities. This lays the foundation for inclusive physical education and participation in sport. Furthermore, Section 38 calls for the adaptation of recreational and sporting programmes to the needs of persons with disabilities, affirming their right to equal participation in social and cultural life. Thus, ensuring that public recreational centres, sport facilities, and cultural venues are accessible to persons with disabilities. Collectively, these provisions position sport as a vital tool for inclusion, rehabilitation, and empowerment, reinforcing the broader rights to education, health, and social integration outlined in the Act.

By obliging the state and other stakeholders to create an enabling environment, Act 715 sets a foundational legal obligation for Ghana to develop inclusive and accessible sport opportunities for persons with disabilities, with implementation responsibilities shared among key state institutions and civil society actors. In light of this, key stakeholders include the Ministry of Gender, Children, and Social Protection (the lead coordinator for disability services), the Ministry of Sports and Recreation (for inclusive sport development), and the Ghana Education Service (which oversees adapted physical education). The National Sports Authority and the NPC-Ghana, along with disability sport federations, are crucial for organising and developing disability sport. At the grassroots level, key stakeholders such as District Assemblies, the DSWCD, Civil Society Organisations, and NGOs are instrumental in providing vital contributions. These contributions include advocating for and empowering persons with disabilities, as well as implementing and funding disability sport programmes.

### 5.4 Sport act

The Sports Act, 2016 (Act 934) came into force, repealing the erstwhile Sports Act, 1976 (SMCD 54). Act 934 establishes the legal and institutional framework for the promotion, regulation, and development of sport in Ghana, explicitly encompassing disability sport. Its formulation was influenced by global trends, including the MDGs, SDGs, and the nascent Sport for Development and Peace (SDP) agenda. Presently, what is lacking, as Charway and Houlihan (19) noted, is a detailed national policy guide, akin to the 1994 National Sport Policy, which provided some (inclusive) depth to the Sports Act, 1976 (SMCD 54). Significantly, the Act 934 establishes a National Sports Authority to develop, promote, and manage sport nationwide, inclusive of disability sport.

Although the Act does not create a distinct category for disability sport, Section 3(m) embeds inclusive provisions that mandate the participation of persons with disabilities in national sport development. Furthermore, in Section 4.1(g)(iii), the Act integrates a representative from the NPC-Ghana onto the governing board of the National Sports Authority and ensures their recognition through the Ministry of Sports and Recreation. In practice, this inclusive governance arrangement has been subject to contestation. Several stakeholders from ASFOD have expressed concerns about being marginalised, arguing that disability sport in Ghana encompasses a broader spectrum of associations beyond the NPC-Ghana, particularly at the community level. From Sections 25–29, the Act also facilitated the creation of Regional Sport Committees and District Sports Units, which are crucial for implementing sport inclusion policies at the grassroots level.

Relevant key stakeholders directly involved in implementing Act 934 include: the Ministry of Sports and Recreation, which administers inclusive sport; the National Sports Authority, responsible for supporting the organisation of disability sport and coordinating with federations; the Ghana Paralympic Committee and Disability Sport Federations, who directly promote and develop disability sport; and the Regional Sport Committees and District Sports Units that implement government inclusive sport policies in local communities. Other key collaborative stakeholders include District Assemblies, the DSWCD, the Ghana Education Service, civil society organisations, and NGOs. All of these bodies complement the Ministry of Sports and Recreation by delivering relevant, inclusive services at the grassroots level.

## 6 Contemporary issues

Despite institutional advances in legitimising and mainstreaming disability sport in Ghana, systemic challenges persist. Key issues include inadequate funding, limited access to training facilities and adaptive equipment, insufficient media coverage/representation, and enduring gender disparities in participation (2, 3). Thus, this indicates that the implementation of Ghana's Persons with Disability Act, 2006 (Act 715), has been slow, particularly concerning sport inclusion. These persistent obstacles continue to impede the full realisation of inclusive sport, notwithstanding the progress achieved thus far. Next, we will discuss current issues affecting the development and organisation of disability sport in Ghana.

### 6.1 Disability sport sustainability in Ghana

Ghana has demonstrated a formal commitment to disability inclusion through several significant legal and policy frameworks. These include the enactment of the Persons with Disability Act, 2006 (Act 715), the signing of the UN-CRPD in 2007, and its subsequent ratification in 2012. These milestones signify the state's recognition of the rights of persons with disabilities, including their right to access and participate in sport and physical activity as enshrined in international and national human rights instruments. This commitment is further reflected in Ghana's alignment with the UN's Agenda for the SDGs, which prominently features disability inclusion. Notably, disability-inclusive provisions appear in SDG targets such as 4.5, 4a, 8.5, 10.2, 11.2, 11.7, and 17.18, and within the Declaration clauses 15, 19, 23, and 60 of the 2030 Agenda (25). Building upon this international commitment, Ghana has endeavoured to institutionalise the UN SDGs through a multi-sectoral approach, integrating various services and a plurality of actors (26). This institutionalisation also reflects how the SDG policy is intended to be implemented at the local level through funding, partnerships, and robust monitoring and evaluation mechanisms.

Furthermore, evidence of the Ghanaian government's commitment, specifically through the Ministry of Finance, is the development of an SDGs budgeting and tracking system. This system serves as a crucial monitoring tool to ensure that budgetary allocations and expenditure across Ministries, Departments, and Agencies (MDAs), as well as district assemblies, align with SDG targets (27). A recent study by Charway et al. (3) found that the Ministry of Sports and Recreation's 2016–2021 Medium-Term Expenditure Framework ostensibly demonstrated a strong track record of prominently featuring and prioritising disability sport programmes organised in various local communities. However, a concerning discrepancy emerges when juxtaposed with the findings from interviews with disabled sport associations conducted by Charway et al. (3), which revealed the opposite. A poignant statement from one disabled sport association member encapsulates this sentiment: “*We feel that they only involve us as a formality and for the books to fulfil their own goals. But when it comes to implementation at any level, we are completely neglected even though we are present everywhere”* Charway et al. (3). This sentiment was corroborated by members of the District Sport Units, who confirmed that they consistently bypass collaborations with disabled sport associations due to severe funding limitations and a pronounced lack of expertise or qualified community coaches capable of effectively including persons with disabilities in district programmes. This dearth of community coaches with specialist competencies fundamentally undermines “full inclusion” (28) as even when District Sports Units are willing, they lack the necessary personnel and appropriate coaching skills.

Moreover, Charway et al.'s (3) audit of the Sports Act, 2016 (Act 934), combined with discussions with District Sport Unit participants, highlighted that despite their presence in various Ghanaian communities, members of disabled sport associations are notably unrepresented within the District Sport Units themselves. This systemic issue is further compounded by the District Sports Units’ assertion that they have not been adequately involved in the institutionalisation of the SDGs within the Ministry of Sports and Recreation, nor have they been properly briefed on its intended implementation at the local level. These findings collectively point to a critical gap between policy aspiration and practical execution, significantly impeding the genuine sustainability of disability sport in Ghana.

### 6.2 Navigating politics and internal conflicts

The evident implementation deficit in Ghana's disability sport sector has given rise to two distinct categories of conflict: firstly, between state sport governing bodies and disabled sport associations, and secondly, among disabled sport associations themselves. The first category of conflict stems from the skewed allocation of support towards amputee football by state entities. It would be unjust to claim that the Ministry of Sports and Recreation, alongside its sub-national structures across Ghana's 16 regions and 261 district assemblies, provides no support whatsoever for disability sport. However, the recurring issue is that when such support is provided, it almost exclusively targets amputee football for the physically challenged. In recent times, the Ministry of Sports and Recreation has notably supported the Ghana national amputee soccer team, managed by the Amputee Football Federation. These forms of support are frequently documented in the Ministry's Medium-Term Expenditure Framework annual reports and are particularly prevalent at the district assembly level, especially during periods of national elections. Charway et al. (29) characterise this as a form of political-symbolic implementation, wherein politicians, both at the ministerial and district assembly levels, deliberately favour football due to its profound historical and cultural heritage, and its immense popularity among Ghanaians. Other disabled sport associations, such as the Ghana Blind Sport Association and the Ghana Deaf Sports Federation, have consistently voiced concerns over the Ministry's Medium-Term Expenditure Framework reports, which often generalise support to all disabled sport associations despite only actively backing amputee football (3, 29).

The second layer of conflict, as previously hinted (under Section 5.4., Sport Act), exists between the NPC-Ghana and ASFOD, where NPC-Ghana has been granted greater recognition and credence in the Sports Act, 2016 (Act 934) over ASFOD. A further complication arises within the blind sport community, specifically concerning the relationship between the Ghana Blind Sport Association and its parent organisation, the Ghana Blind Union. Established in April 2010 through a merger of the Ghana Society for the Blind and the Ghana Association of the Blind, the Ghana Blind Union has been instrumental in supporting, resourcing, and enhancing the community presence of the regional sport-wing concept, which serves as an extension of the Ghana Blind Sport Association. However, much like the Ghana Deaf Sports Federation's independence from the Ghana National Association of the Deaf, the Ghana Blind Sport Association desires autonomy from the Ghana Blind Union. This pursuit of independence has proven challenging, leading to threats from the erstwhile leadership of the Ghana Association of the Blind to form a new blind sport association. Their stated objectives are to alleviate bureaucratic burdens, reduce the duplication of reporting to both the Ghana Blind Union and the NPC-Ghana, and ultimately foster greater autonomy within the blind sport sector. These internal frictions underscore the complex political dynamics and governance challenges impeding a more unified and equitable development of disability sport across Ghana.

### 6.3 Quest for international recognition amidst visa racketeering

A striking paradox characterises Ghana's approach to disability sport—a sector consistently starved of grassroots and elite development funding suddenly attracts governmental interest when its national teams achieve qualification for international championships. This sporadic attention, however, often conceals a more insidious issue which include the exploitation of disabled sport through systemic visa racketeering. Such illicit practices severely erode athlete confidence and gravely tarnish the image and credibility of Ghanaian disability sport associations (Koranteng, 2024).

Two prominent incidents have brought this issue to global attention. In July 2009, state officials accompanying the Ghanaian Deaf Football team to a match against Deaf Australia were implicated in facilitating the inclusion of 25 non-deaf individuals within a 30-player contingent (30). More recently, on 27th April 2024, a highly publicised event saw an 11-member team, including purported para-athletes (later identified by the NPC-Ghana as “fake”), abscond upon arrival in Norway for the Bergen Marathon. Tragically, one individual subsequently died, whilst another was apprehended attempting to enter Sweden. The deliberate deception inherent in presenting non-genuine para-athletes profoundly damages Ghana's international standing. Although visa applications for international para-sport competitions can sometimes proceed without official endorsement from both the Ministry of Sports and Recreation and the NPC-Ghana, the recurring involvement of officials from these very organisations in such incidents, coupled with their consistent denials of culpability, raises serious questions regarding accountability. The unfortunate consequence of these fraudulent schemes is that genuine para-athletes are routinely denied the opportunity to represent their nation with pride. As lamented by Frederick Assor, Athlete Commissioner on the NPC-Ghana Board, in a 2024 radio interview, “*We’ve seen that it's still happening. So, we are here once again to draw the Ministry's attention that we persons with disabilities are being used […] for visa racketeering here and there. We just see our names, our events, being used to travel out of the country, whilst we are not aware. And it is tarnishing the image of we persons with disability*” (31).

These repeated scandals have significant implications for disability sport in Ghana. Firstly, they severely undermine trust among athletes, within disability sport associations, and critically, between these organisations and state bodies. Consequently, international bodies and potential sponsors may become increasingly hesitant to invest in Ghanaian disability sport if funds are perceived to be vulnerable to corrupt practices. Secondly, the persistent denials of culpability by the Ministry and NPC-Ghana, despite evidence of official involvement, underscore significant governance and accountability deficits within Ghana's sport administration. This necessitates thorough and transparent investigations, with clear lines of responsibility established and appropriate sanctions applied to deter future occurrences. Furthermore, if such controversies taint elite representation, it sends a negative message to grassroots initiatives, diminishing the incentive to develop talent at the community level if the pathway to international competition is perceived as compromised by illicit activities. Finally, these incidents, often involving the fraudulent misrepresentation of individuals as disabled athletes, inadvertently reinforce negative stereotypes, potentially hindering broader societal inclusion efforts for persons with disabilities.

Addressing this systemic issue demands a multi-faceted approach. This includes implementing stringent visa application protocols, enhancing oversight mechanisms within both the Ministry of Sports and Recreation and the NPC-Ghana, ensuring transparent accountability measures, and demonstrating an unwavering commitment to prosecuting those involved in visa racketeering. Adhering to these decisive actions, Ghana can restore integrity to its disability sport landscape and ensure that genuine athletes receive the support and opportunities they rightfully deserve.

### 6.4 Socio-structural barriers and gender dynamics

Extensive scholarship on the experiences of persons with disabilities in Ghana consistently highlights pervasive issues of stigma, social exclusion, and discrimination across various domains, including health, employment, and daily life. These challenges are often deeply influenced by unspoken African norms and ingrained myths (32–35). Within the realm of sport, Charway et al. (3) identify a similar pattern of socio-structural barriers. Their research reveals that deliberate neglect and discrimination, coupled with a notable lack of representation for persons with disabilities within District Sport Units, create significant challenges for both persons with disabilities and the broader development of disability sport. Beyond these socio-structural barriers lies the critical issue of media coverage and its relationship to social justice. Although the Ghanaian media generally demonstrate a willingness to cover disability issues, including sport, Amoako et al. (36) compellingly argue that “in a neo-liberal society [like Ghana], the media seems to have sidestepped social justice in favour of profitability.” This observation resonates acutely with disability sport in Ghana. Disability sport rarely features prominently in daily radio, print media or online programming, and regrettably, its visibility is often amplified only when associated with negative press, such as the widely publicised visa racketeering and “fake athlete” representation scandals. This selective and sensationalist coverage further marginalises a sector already struggling for recognition.

Another significant area that has received insufficient scholarly attention, particularly within the Ghanaian context, is the complex interplay between gender dynamics and participation in disability sport. While not its primary focus, Bourgeois' (2) article, “Contested perspectives of ‘marvel’ and ‘mockery’ in disability and sport,” in Ghana offers valuable insights. Bourgeois (2) asserts that although “disabled women and men face many challenges […] women with disabilities have somewhat different, and markedly more, worries than men with disabilities, as manifested ‘off-court,’ primarily in terms of relationships, marriage, education and employment” (p. 1247). Concurrently, Bourgeois observed that men also confront unique difficulties, referring to their situation as “doubly bound” due to societal expectations of hegemonic masculinity which are challenging to meet given their physical limitations, Bourgeois (2). While Bourgeois' research is not recent, it critically illuminates gendered challenges that remain largely under-explored in the discourse on disability sport in Ghana.

Considering these deeply entrenched socio-structural challenges, the limited and often negatively skewed media coverage, and the under-examined gender dynamics, it becomes evident that disability sport in Ghana presents a complex conundrum, demanding further scholarly investigation to fully unpack its multifaceted challenges.

## 7 Concluding discussion

This discussion illuminates the contextualised barriers that persons with disabilities continue to encounter in Ghana. Acknowledgment of these barriers by both society and government necessitates a concerted effort from state and non-state actors to significantly advocate for their removal, thus enabling the genuine inclusion of persons with disabilities in mainstream provisions. Through this article, the authors have traced the historical evolution of disability sport in Ghana, revealing shifting attitudes towards persons with disabilities through various lenses. Indigenous cultural beliefs, African traditional religions, foreign religious beliefs, and colonial practices have all negatively impacted attitudes towards people born with disabilities and those who acquire them later in life. As the Ghanaian society progressed through different periods (pre-colonial, colonial, and post-colonial) a gradual shift towards inclusive participation for disabled members of society is noted. However, these changes emerged through diverse agentic movements within society and external influences, demonstrating a political commitment to international conventions such as the UN-CRPD, ratified in 2012.

Although there has been notable progress in legislative frameworks, fragmentation appears to exist in the roles undertaken by different entities responsible for disabled affairs. Such a disconnect can, in some instances, lead to conflicting aspirations between state and non-state actors, as well as among non-state actors themselves. The complexities arising from the overlapping jurisdictions of each state and non-state organisation may be perceived as limiting the advancement of positive initiatives aimed at addressing exclusionary tendencies affecting persons with disabilities. To effectively tackle the systemic issues restricting the inclusive participation of persons with disabilities, clarity of roles and the public value of these roles are essential to ameliorate barriers and promote genuine inclusive participation. The authors emphasise that the exclusion of entities directly representing those impacted is a recipe for failure in developing authentic solutions to address the diverse needs and realities of the disability sport community.

For instance, the authors have highlighted the marginalisation of both grassroots organisations, such as the ASFOD, and the limited representation of persons with disabilities within decision-making forums like the District Sports Units. This underscores the critical need to uphold the adage popular within disability rights literature advocating for the inclusion and self-determination of persons with disabilities: *nothing for us without us* (37). There is a pressing need to ensure authentic, rather than tokenistic, engagement of persons with disabilities in decision-making processes concerning their affairs. These exclusionary tendencies are not solely confined to institutional hurdles but also emanate from traditional cultural practices that perpetuate ingrained stigmatisation and discrimination against persons with disabilities. Ghana is no exception, as these practices are widespread in various societies worldwide.

Despite the persistent challenges hindering the inclusive implementation of disability sport-focused initiatives, the authors strongly believe that there is significant policy attention directed towards disability sport in Ghana. This is evidenced by the diverse range of legal and policy frameworks that have been developed or adopted to advance disability sport, including the Sports Act, 2016 (Act 934). It is arguably beneficial to have these frameworks in place, even with limited economic capital to fully resource implementation plans. Chronic failures in the implementation of disability sport initiatives have been fundamentally linked to inadequate funding and unsuitable equipment for programme execution.

Furthermore, the self-interest of political actors has demonstrated a bias towards amputee football, often at the expense of distributing resources to meet the diverse sporting and leisure needs of broader disability sport communities. Such tendencies, where amputee football is used for political gain during campaigns, inadvertently limit efforts for holistic development by state and non-state organisations like the National Sports Authority or the NPC-Ghana, respectively. Other notable challenges include limited and often negatively skewed media coverage, where the media has at times focused on negative aspects rather than celebrating Ghanaian disability sport achievements. Equally, systemic visa racketeering practices, involving fraudulent misrepresentation of athletes, have caused global reputational damage for Ghana's disability sport community. This requires decisive action from state actors and the NPC-Ghana to eradicate these practices.

Recommendations for disability sport in Ghana emphasise moving beyond using amputee football for political gain and instead spreading attention across all disability sport disciplines. The article highlights the complexities in governance due to the congested landscape of disability sport representative bodies. Despite this, inclusive governance is particularly lacking at the grassroots level concerning representation in decision-making within the District Sports Units. There is a clear need to promote and fully integrate representative disability sport bodies whose public value enhances inclusive participation to meet the diverse needs of the Ghanaian disabled community.
