# Correction: Disability sport profile of Ghana: evolution, policies, politics and participation barriers

**DOI:** 10.3389/fspor.2026.1917783

**Published:** 2026-07-13

**Authors:** Derrick Charway, Dennis Osei-Nimo Annor, Davies Banda

**Affiliations:** 1 Department of Sport and Social Sciences, Norwegian School of Sport Sciences, Oslo, Norway; 2 Health and Physical Cultures for Social Justice, Western University, London, ON, Canada; 3 Moray House School of Education and Sport, ISPEHS, The University of Edinburgh, Edinburgh, United Kingdom

**Keywords:** ableism, barriers, Ghana, mainstreaming disability sport, policy implementation

Affiliation 1, “Department of Sport and Social Sciences, Norwegian School of Sport Sciences, Oslo, Norway”, was erroneously given as “Department and Sport and Social Sciences, Norwegian School of Sport Sciences, Oslo, Norway”.

The original version of the article has been updated.

